# Characterization of graded 6-Hydroxydopamine unilateral lesion in medial forebrain bundle of mice

**DOI:** 10.1038/s41598-024-54066-0

**Published:** 2024-02-14

**Authors:** Juntao Cui, Di Zhao, Manman Xu, Zheheng Li, Junliang Qian, Ning Song, Jun Wang, Junxia Xie

**Affiliations:** 1 Institute of Brain Science and Disease, Qingdao University, Qingdao, 266071 China; 2 Shandong Provincial Key Laboratory of Pathogenesis and Prevention of Neurological Disorders, Qingdao University, Qingdao, 266071 China; 3 School of Basic Medicine, Qingdao University, Qingdao, 266071 China

**Keywords:** Parkinson’s disease, 6-Hydroxydopamine, Medial forebrain bundle, Motor behavior, Non-motor behavior, Gait analysis, Glial cell activation, Mitochondrial dysfunction, Neuroscience, Physiology, Medical research

## Abstract

Parkinson’s disease (PD) is the second most common age-related neurodegenerative disease, with a progressive loss of dopaminergic cells and fibers. The purpose of this study was to use different doses of 6-hydroxydopamine (6-OHDA) injection into the medial forebrain bundle (MFB) of mice to mimic the different stages of the disease and to characterize in detail their motor and non-motor behavior, as well as neuropathological features in the nigrostriatal pathway. MFB were injected with 0.5 μg, 1 μg, 2 μg of 6-OHDA using a brain stereotaxic technique. 6-OHDA induced mitochondrial damage dose-dependently, as well as substantia nigra pars compacta (SNpc) tyrosine hydroxylase-positive (TH^+^) cell loss and striatal TH fiber loss. Activation of astrocytes and microglia in the SNpc and striatum were consistently observed at 7 weeks, suggesting a long-term glial response in the nigrostriatal system. Even with a partial or complete denervation of the nigrostriatal pathway, 6-OHDA did not cause anxiety, although depression-like behavior appeared. Certain gait disturbances were observed in 0.5 μg 6-OHDA lesioned mice, and more extensive in 1 μg group. Despite the loss of more neurons from 2 μg 6-OHDA, there was no further impairment in behaviors compared to 1 μg 6-OHDA. Our data have implications that 1 μg 6-OHDA was necessary and sufficient to induce motor and non-motor symptoms in mice, thus a valuable mouse tool to explore disease progression and new treatment in PD.

## Introduction

Parkinson's disease (PD) is the second most common age-related neurodegenerative disease, affecting approximately 6 million people worldwide^1,2^. The motor symptoms of PD are bradykinesia, resting tremor, rigidity and postural instability. The classical concept is that the first motor symptoms of PD usually appear in patients when approximately 60% of the substantia nigra pars compacta (SNpc) dopamine (DA) neurons have degenerated and 70–80% of the normal striatal DA level is lost^3–5^. The neuropathological hallmarks of PD are loss of substantia nigra dopaminergic neurons, decreased striatal DA release and the concomitant presence of cytoplasmic inclusions or lewy bodies composed primarily of alpha-synuclein. In addition, non-motor symptoms such as anxiety, depression, sleep disturbance, intestinal dysfunction and cognitive decline are increasingly considered to be the main determinants of patient quality of life^6,7^. Non-motor symptoms of PD can occur at all stages of the disease, and some non-motor symptoms, such as hyposmia, abnormal rapid eye movement sleep behavior, constipation, and depression, may occur earlier than motor symptoms^8^. Non-motor symptoms may also fluctuate with movement fluctuations. However, the etiology of PD is unclear, and multiple mechanisms have been implicated in the pathogenesis of PD, including oxidative stress, mitochondrial dysfunction, protein misfolding/aggregation, autophagy, neuroinflammation, defects in the neurotransmitter system, and excitotoxicity.

The development of animal models of PD using genetic and toxin-based approaches is improving our understanding of the pathophysiology of the disease and providing new experimental tools for testing new therapies. Patients with PD often present with asymmetric symptomatic episodes affecting only one side of the body, and the establishment of preclinical models that mimic this aspect is important for the evaluation of any new therapeutic approach^9^. 6-OHDA, a catecholamine-selective neurotoxin, causes mitochondrial respiratory dysfunction via the dopamine (DA) reuptake transporter, leading to oxidative stress-induced toxicity and neuroinflammation, ultimately resulting in cell death^10,11^. The most commonly used animal model of PD is the unilateral injection of 6-OHDA into the medial forebrain tract (MFB), which leads to complete denervation of the dopaminergic neuronal pathway in the substantia nigra and striatum to induce motor damage and cause asymmetric motor symptoms^12^. The unilateral 6-OHDA rat lesion model has been widely used to model PD and potential therapeutic approaches, but relatively few studies have been performed in mice. In mice, 6-OHDA has been stereotactically injected into different brain regions along the nigrostriatal pathway, such as the striatum^13–15^, SNpc^16^, and MFB^17,18^, resulting in different types of high mortality rate^19^, and differences in 6-OHDA injection dose, injection volume, injection speed and injection coordinates. However, this can be overcome with appropriate postoperative care. When injected into the MFB or SNpc, 6-OHDA induced more complete and faster lesions in the nigrostriatum, with neurons beginning to degenerate within 24 h^20^. When injected into the striatum, it induced slow and sustained degeneration, resulting in a more gradual and stable loss of DA neurons^19^. An advantage of injecting 6-OHDA into the MFB is that the toxin is not injected into the striatum or SNpc, where cell transplantation and potential therapeutic approaches such as converting glial cells into neurons can take place^21–23^.

Gait disturbances are one of the most common motor problems in PD. Clinically, patients with PD are characterized by gait disturbances such as short, shuffling steps with freezing episodes, reduced overall speed, reduced arm swing, shorter step length, shorter stride length and increased duration of the stance phase^24–26^. When investigating multifaceted and complex behaviors such as gait, it is essential to use an appropriate method to assess changes. In recent years, a novel automated quantitative gait analysis method, the CatWalk, has been introduced to provide automated and simultaneous quantification of many static and dynamic aspects of gait during voluntary walking. This method has previously been evaluated for use in animal models of pain^27^, spinal cord injury^28^ and arthritis^29^. Several studies have shown Catwalk to be a reliable tool for assessing gait in rodent models of many neurodegenerative diseases, including PD^30,31^. In the past, gait analysis of 6-OHDA injury has mainly focused on rats, including drug intervention, brain stimulation, and cell transplantation to improve gait^30,32,33^. In mice, despite a study on gait analysis, 50% of mice withdrew from their protocol^34^.

In the present study, we established MFB injury models in mice at different doses (volume: 1 μl, 6-OHDA concentration: 0.5 μg/μl, 1 μg/μl, 2 μg/μl)^35,36^. By investigating which doses of 6-OHDA produced predictable levels of partial nigrostriatal lesions, it was associated with significant behavioral deficits. We assessed the loss of TH^+^ cells and TH fibers associated with different doses of 6-OHDA, and examined dose-dependent motor symptoms (including gait changes), non-motor symptoms (anxiety-like behaviors, depression-like behaviors), mitochondrial dysfunction and glial cell activation.

## Methods

### Mice

Male C57BL/6 J mice (8 weeks old) were obtained from Beijing Vital River Laboratory Animal Technology Co., Ltd. All animals were acclimatised for 1 week in housing of 5 animals per cage, with free access to food and water, and in rooms maintained on a 12-h light/dark cycle. All experiments were performed during the light period.

### Unilateral MFB 6-OHDA lesion surgery

Thirty minutes before surgery, each animal should be weighed and the weight recorded. Systemically administer desipramine hydrochloride (2.5 mg/ml, Sigma Aldrich) and pargyline hydrochloride (0.5 mg/ml, Sigma Aldrich) (0.9% sterile saline, pH 7.4) at 10 ml/kg by intraperitoneal injection (i.p) to one mouse using a 1 ml syringe. For example, a 23.0 g mouse would receive 230 μl of premedication. Thirty minutes after administration of the desipramine HCl and pargyline HCl solutions, the mouse is placed in a closed anaesthesia chamber and anaesthetised by inhalation of isoflurane (2–3% in O_2_). The animal is sufficiently anaesthetised when it shows no response to hind limb pinch and no blink reflex. The top of the mouse's head is shaved and the iodophor is applied directly to the skin using cotton wool to sterilise the animal's head. The mice were placed in an appropriate stereotactic frame. First, they were placed in the incisor bar and then the anaesthetic mask was placed on their face. The oxygen flow was then adjusted to 1 L/min and the isoflurane to 1.5–2%. The incisor bar is inserted into the earmuff when it is in a horizontal position relative to the earmuff. If the head is completely flat, the earmuff is correctly inserted. The screws are tightened and cannot move in either direction. The skull has been cleaned with 3% oxygen and the bregma exposed.

Mice received a 1 μl injection of 6-OHDA hydrobromide (Sigma Aldrich, Australia) in 0.02% ascorbic acid into the left MFB at the following coordinates: AP -1.2, ML -1.2, DV -5.0, relative to bregma and the dural surface. The following 6-OHDA concentrations were used in this study: 0.5 μg/μl, 1 μg/μl, and 2 μg/μl. The sham group was injected with 0.02% ascorbate/saline solution and used as a control. The injection rate of 6-OHDA (or 0.02% ascorbic acid control) was 0.2 μl/min and the syringe was left in place for a further 10 min after injection to allow complete diffusion into the target area. The needle was slowly withdrawn. The incision was sutured and the animals were placed in individual cages on heating pads. To minimise mortality, special post-operative care was provided for the first two weeks after surgery. During the postoperative recovery period, the mice were fed wet chow to ensure adequate nutrition. A common post-operative complication is dehydration, as evidenced by slow retraction of the skin after skin pinching. If this is observed, 1 ml of lactated Ringer's solution should be given subcutaneously for 1 week or until symptoms improve.

### Behavioural tests

**Figure 1:**
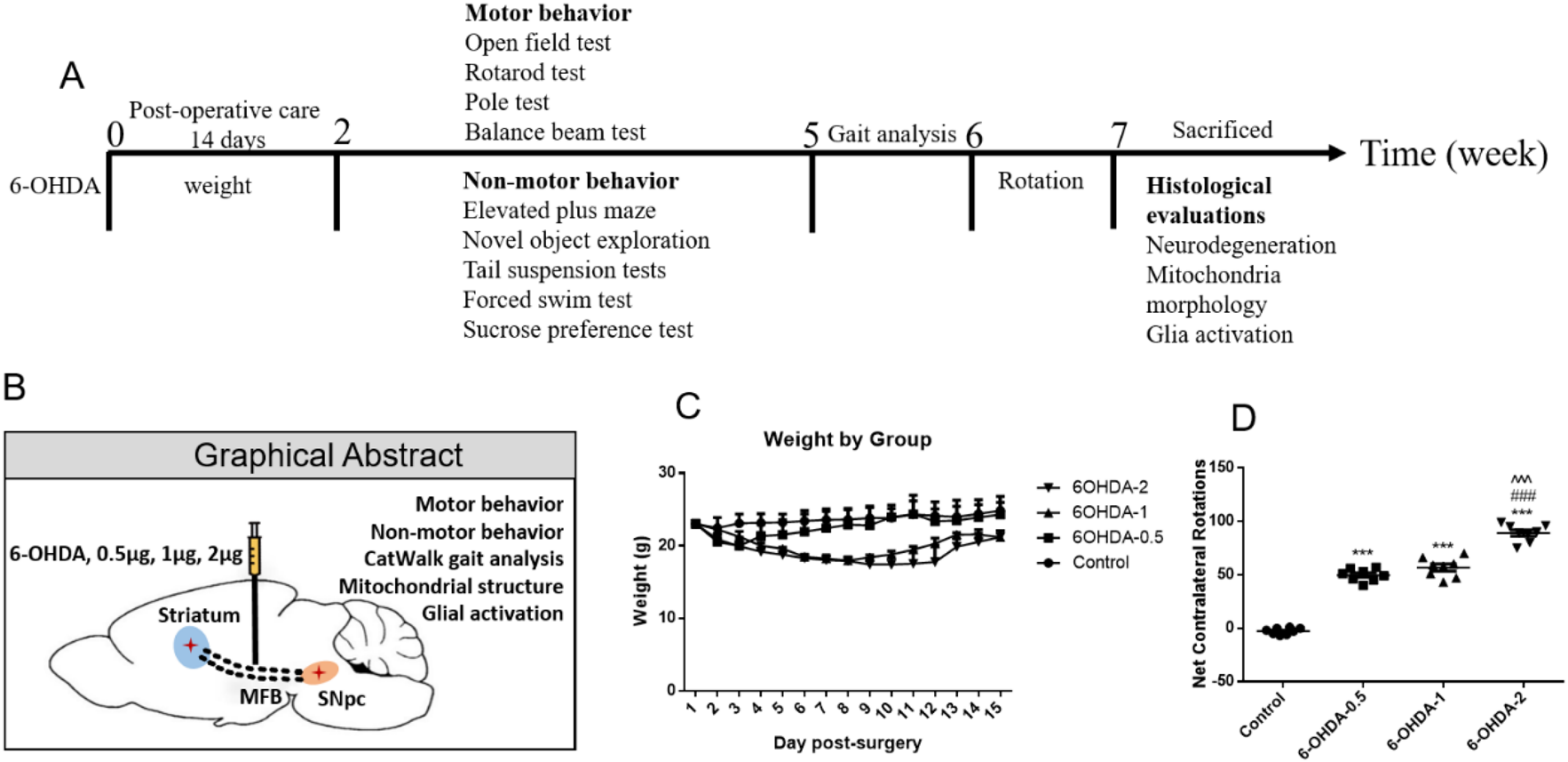
Experimental design, graphical abstract, body weight changes and apomorphine-induced rotation. Time sequence of mice receiving different behavioral tests and other tasks (**A**). Body weight for the 14 consecutive days post lesion plotted per group (**C**). Apomorphine induced the turning behavior of animals with different doses of 6-OHDA (**D**). ***P < 0.001 compared with control, ^###^*P* < 0.001 compared with 6-OHDA-0.5, ^^^^^*P* < 0.001 compared with 6-OHDA-1.

Behavioural assessments were performed fourteen days after the surgery when mice achieved a complete post-surgery recovery. The behavioural tests avoiding more than one test per day and starting with the non-drug behavioural tests, followed by the drug-induced behavioural tests. Prior to each behavioural test, animals were habituated to the test room for 2 h. Behavioural tests and graphical abstract were conducted in the order outlined in Fig. 1A,B and as described below.

### Open field test

Open field test is a classic emotion related behavioral test, which is used to detect the behavior of animals in unfamiliar environments, including spontaneous activity behavior and exploration behavior^37^. It is a method to evaluate the autonomous behavior, exploration behavior and tension of animals in new and different environments. Animals were placed at the center of an illuminated square arena (40 cm × 40 cm), enclosed by a 40 cm high wall. The distance of the central area is 20 cm × 20 cm. The animals shall be placed in the center of the test box from the same position and direction during each experiment. The animals were put into the test box to adapt for 1 min, and the test time was 10 min. The animal movement track was recorded by automatic camera and analyzed by behavioral analysis software (intelligent video tracking system V3.0 smart 3.0). The detection indicators were movement distance, immobility time and central area residence time.

### Rotarod test

The rotarod test is based on the performance of the long cylindrical rotating rod, which requires the animals to have complete motor skills and sensory motor integration^38^. The residence time of the animals on the rotating rod can be used as an indicator to measure the motor function of experimental animals. This experiment can be used to evaluate the balance, grip and motor coordination of rodents. The mice were trained three per day over two consecutive days separately, at 11 rpm per minute on the first day and 22 rpm per minute on the second day. Each training session lasted 300 s, with an interval of at least one hour to prevent fatigue. On the test day, the rod was programmed to accelerate from 4 to 40 rpm in 300 s. The time and speed of falling from the rotating rod are recorded, and the average value of three times is used for statistical analysis.

### Balance beam test

The balance beam test was used to analyze the sensorimotor function of rodents^39^. In the first two days, the mice were trained to cross the beam until they could cross the entire distance of the instrument by first accustoming them to the target box and then moving them to the starting point of the continuous test farther away from the entrance of the target box. On the third day (test day), each mouse was videotaped in three consecutive trials, scored and counted according to Feeney's improved rating scale.

0 score, the mice failed stay on the balance beam and directly fall. 1 scores, the mouse does not move but can stay on the balance beam. 2 score. The mouse tried to cross the balance beam but failed. 3 score: the mouse can climb over the balance beam, but the number of times that its damaged contralateral hind limb slides down the balance beam is more than 50%. 4 score, the mice can climb over the balance beam, and the number of times that their damaged contralateral hind limbs slide down the balance beam is less than 50%. 5 score, the mice can climb over the balance beam, and the number of times that the hind limbs on the opposite side of the damaged side slide down the balance beam is only once. 6 score, the mouse can climb over the balance beam, and the damaged contralateral hind limbs will not slide down the balance beam.

### Pole test

The pole test was used to evaluate the motor retardation in mice^40^. Place the mouse head up on the top of a rod with a vertical rough surface (diameter: 8 mm; height: 50 cm), and record the time when it drops to the ground. The maximum duration is 120 s. Even if the mouse dropped a part, and dropped in the rest, its behavior was scored until it reached the ground. When the mouse could not rotate downward but fell from the pole, the descent time was counted as 120 s due to serious injury. The mice were trained three times a day for two consecutive days. On the experimental day, the average value of the three times was used for statistical analysis.

### Elevated plus maze

The elevated plus maze test, which is widely used for anxiety and its related symptoms, takes advantage of the rodent's dislike of open spaces and seeks shelter as much as possible^41^. Without any acclimation or training prior to the elevated plus maze, the mice were removed from their cages and placed gently on the central area of the apparatus facing the open arms. Open the behavior analysis software, set the parameters, and start the detection after 1 min of adaptation. The detection time is 5 min. Animal movement trajectories were recorded by automatic cameras and analyzed by behavior analysis software (intelligent video tracking system V3.0 smart 3.0). The detection indicators are the moving distance, the dwell time in the center area, and the number and time percentage of entering the open arm.

### Novel object exploration

Novel object exploration is to study the exploration behavior of animals to novelty, which is based on the innate novelty-seeking behavior of animals, and use this method to assess whether animals are depressed^42^. The novel object exploration is divided into two stages: adaptation period and test period. The operation steps are as follows: (1) Adaptation period: The animals are put into the spontaneous activity test box and adapted for 5 min. (2) Test period: Introduce a new object (should be placed in the center position) under the same environmental conditions, and record the number of explorations and the exploration time of the new object within 10 min. Animal movement trajectories were recorded by automatic cameras and analyzed by behavior analysis software (intelligent video tracking system V3.0 smart 3.0). The criteria for judging exploratory behavior are: the distance between the snout and the nose of the animal is less than 2 cm from the novel object or the object is in direct contact with the object.

### Tail suspension test

The tail suspension experiment is to hang the tail of the mouse, and the head of the mouse is in an upside-down position. At first, the mouse will struggle violently to try to escape this uncomfortable state, but after struggling for a period of time, it is found that there is no hope of escape and it will show a state of immobility, also considered a desperate state^43^. In the mouse tail suspension experiment, 3/4 of the mouse tail was fixed on a hook, about 30 cm above the ground, and the camera was placed horizontally with the mouse suspension device. Animal behaviours were videotaped from the side. The experiment time was 6 min, and the immobile time of the animals within 4 min was recorded. Animal movement trajectories were recorded by automatic cameras and analyzed by behavior analysis software (intelligent video tracking system V3.0 smart 3.0). The detection index was the percentage immobility time.

### Forced swim test

The forced swimming experiment is to force animals to swim in a confined space where they cannot escape. Because rodents have an innate aversion to water, they will struggle to swim in the water and try to escape from the water environment. After a period of time, they find that there is no hope of escape. When the animal stops struggling, it exhibits a state of despair (immobility), which is one of the most commonly used assays for the study of depressive-like behavior in rodents^44^. Animals were placed individually in a cylinder with water temperature of 23–25 °C and water depth set to prevent animals from touching the bottom with their tails or hind limbs. The experiment time was 6 min, and the immobile time of the animals within 4 min was recorded. Immobile time was defined as time when animals remained floating or motionless with only movements necessary for keeping balance in the water. Animal movement trajectories were recorded by automatic cameras and analyzed by behavior analysis software (intelligent video tracking system V3.0 smart 3.0). The detection index was the percentage immobility time.

### Sucrose preference test

The sucrose water preference test is a classic test to detect anhedonia, a typical symptom of depression^45^. During the 48 h water training, 2% sucrose water and drinking water should be given throughout the whole process (the positions of the two water bottles were exchanged in the middle). After the training period, animals were deprived of water and food for 24 h and then exposed to a bottle of 2% sucrose and a bottle of water for 24 h during the dark phase. Switch bottle position after 12 h. The total consumption of each liquid was measured, and the sucrose consumption rate was calculated by dividing the total consumption of sucrose by the total consumption of water and sucrose.

### Rotation test

Drug-induced rotation has previously been validated in the lesion mouse model^46^. Apomorphine-induced rotation test was performed to study the hypersensitivity of the lesioned striatum, assessed by injecting 0.5 mg/kg of apomorphine s.c. (dissolved in a 0.2 mg/mL ascorbic acid in 0.9% saline solution). Each mouse was placed in a glass cylinder and was videotaped using an overhead camera. Full turns were counted in the ipsilateral and contralateral directions during a 20 min window of peak rotational response and data are expressed as net rotations (5–25 min after injection of apomorphine hydrochloride.

### Gait analysis

Mice were subjected to gait assessment with the CatWalk automated gait analysis system (Noldus Information Technology, Wageningen, Netherlands), according to manufacturer’s instructions. The apparatus contains a long glass plate as a runway for animals. When animals traverse from one side of the glass plate to the other, their footsteps are illuminated by fluorescent light emitted from below. The illuminated footsteps are recorded by a high-speed camera installed underneath the glass plate. Each mice underwent pretraining to pass through the Catwalk glass plate within 10 s without stopping. Three runs per trial were recorded and analyzed by Catwalk XT 10.0 gait analysis software. Data acquisition took place in a darkened room with the same researcher handling each subject. Trials in which the animal stopped partway across or turned around during a run were excluded from analysis. A minimum of three valid runs, or complete walkway crossings, were obtained for each subject and the statistics of multiple parameters, such as general gait parameters (run duration, run average speed, cadence, and number of steps), static gait parameters (stride length, print area, max contact area and base of support), dynamic gait parameters (stand, swing duration, swing speed, step cycle, stand index and body speed).

### Transmission electron microscopy (TEM)

The mice were anesthetized with pentobarbital sodium and quickly infused with 0.9% normal saline, followed by 2% PFA and 2.5% glutaraldehyde. The brain was isolated and the striatum/ SNpc of the mouse brain was sliced into 1mm^3^ slices using a horizontal vibrating microtome and fixed in 2.5% glutaraldehyde. After sectioning and double staining with uranium lead, the specimens were observed and photographed by TEM system (JEM-1200, JEOL, Japan).

### Immunohistochemistry and immunofluroescense

After completion of behavioural testing the mice were terminally anaesthetised with sodium pentobarbital and transcardially perfused with 0.9% saline, followed by ice-cold 4% paraformaldehyde (PFA) (dissolved in 0.1 M phosphate buffer, pH 7.4). Brains were rapidly extracted and post-fixed in the same fixative solution for 6 h, and then cryoprotected in 30% phosphate-buffered sucrose (in 0.1 M PBS) overnight. Coronal sections of 30 μm thickness were cut on a freezing microtome and representative sections of striatum, nucleus accumbens (NAc), SNpc and ventral tegmental area (VTA) were selected for free-floating immunohistochemistry to visualize dopaminergic innervation. For immunohistochemistry, the sections were fixed with 4% PFA for 10 min, washed with 0.3% Triton X-100 in 0.01 M PBS (PBS-T) for 10 min (3 times), and sealed in goat serum for 2 h. They were then incubated with polyclonal rabbit anti-mouse tyrosine hydroxylase (TH, 1:2000; AB152, Merck) diluted in the same blocking solution as described above, overnight at 4 °C. After incubation with the primary antibody, rinsed sections with 0.3% PBS-T for 10 min (3 times) and incubated with the goat anti-rabbit IgG-horseradish peroxidase secondary antibodies (Absin, Shanghai, China, 1:500) for 2 h at room temperature. After washing, immunolabeling was detected with 3,3′-Diaminobenzidine (DAB) according to manufacturer’s instructions. The number of TH^+^ cells in SNpc was determined by unbiased stereology counting. The medial boundary of SNpc and the lateral boundary of the VTA are defined by a vertical line passing through the medial tip of the stalk. Data are presented as the percentage (%) compared to the control group. Use Image J software (National Institutes of Health) to measure in all sections, divide the whole striatum into two equal parts along the dorsal and ventral axis, and correct the measured value by non-specific background staining by subtracting the value obtained from the corpus callosum^47^. Data are presented as the percentage (%) compared to the Control group.

To detect glial fibrillary acidic protein (GFAP), ionized calcium-binding adaptor 1 (Iba-1) and TH sections were first fixed in 4% PFA for 10 min, then rinsed in 0.3% PBS-T for 10 min (3 times), and then Blocked with PBS-T containing 0.5% donkey serum for 2 h, sections were finally diluted with 3% PBS-T in the following primary antibodies: mouse anti-GFAP (1:300, 3670S, CST), anti-Iba-1 rabbit (1:200, 19198S, CST) and anti-TH chicken (TH, 1:2000; ab76442, abcam), incubate overnight at 4 °C. The next day, sections were rinsed in 0.3% PBS-T for 10 min (3 times), then sections were diluted with the following secondary antibodies (Alexa Fluor: 647 donkey anti-mouse, 647 donkey anti-rabbit, 488 donkey anti-chicken; ThermoFisher Scientific) in 3% PBS-T incubate for 2 h at room temperature. Nuclei were stained with 4–6-diamidino-2-phenylindole hydrochloride (DAPI, Biyuntian) for 10 min at room temperature. Sections were rinsed in 0.3% PBS-T for 10 min (3 times), mounted with 70% glycerol, and further observed under a fluorescence microscope. Data are presented as the percentage (%) compared to the control group, as measured in all sections using image J software (National Institutes of Health).

### Statistical analysis

All data are expressed as mean ± standard deviation (SD) and were analyzed using GraphPad Prism version 6.0 software (GraphPad Software Inc., San Diego, CA, USA). Statistical analyses were performed by comparing the means of different groups using one-way ANOVAs with Tukey’s multiple comparisons test. Data with glia activation were analysed using independent student’s t-test. with *P* < 0.05 considered significant.

### Ethical approval

All animal work has been conducted according to relevant national and international guidelines and in compliance with the animal Research: Reporting of In Vivo Experiments (ARRIVE) guidelines. Procedures were carried out in accordance with the NIH Guide for the Care and Use of Laboratory Animals and were approved by the Ethical Committee of the Medical College of Qingdao University.

## Results

### Nigrostriatal system histological characterization of 6-OHDA lesions

After 14 days of post-operative care, all mice recovered from surgery and their weight returned to normal level (Fig. 1C). During this period, moist food shall be provided twice a day to monitor and prevent dehydration. Apomorphine, a DA receptor agonist, is the most commonly used test to evaluate the unilateral lesion of 6-OHDA, which was performed to assess the functional integrity of the dopaminergic system. As expected, there was a dramatic increase of rotations in 0.5 μg and 1 μg groups, and even more remarkable in 2 μg group (Fig. 1D).

**Figure 2:**
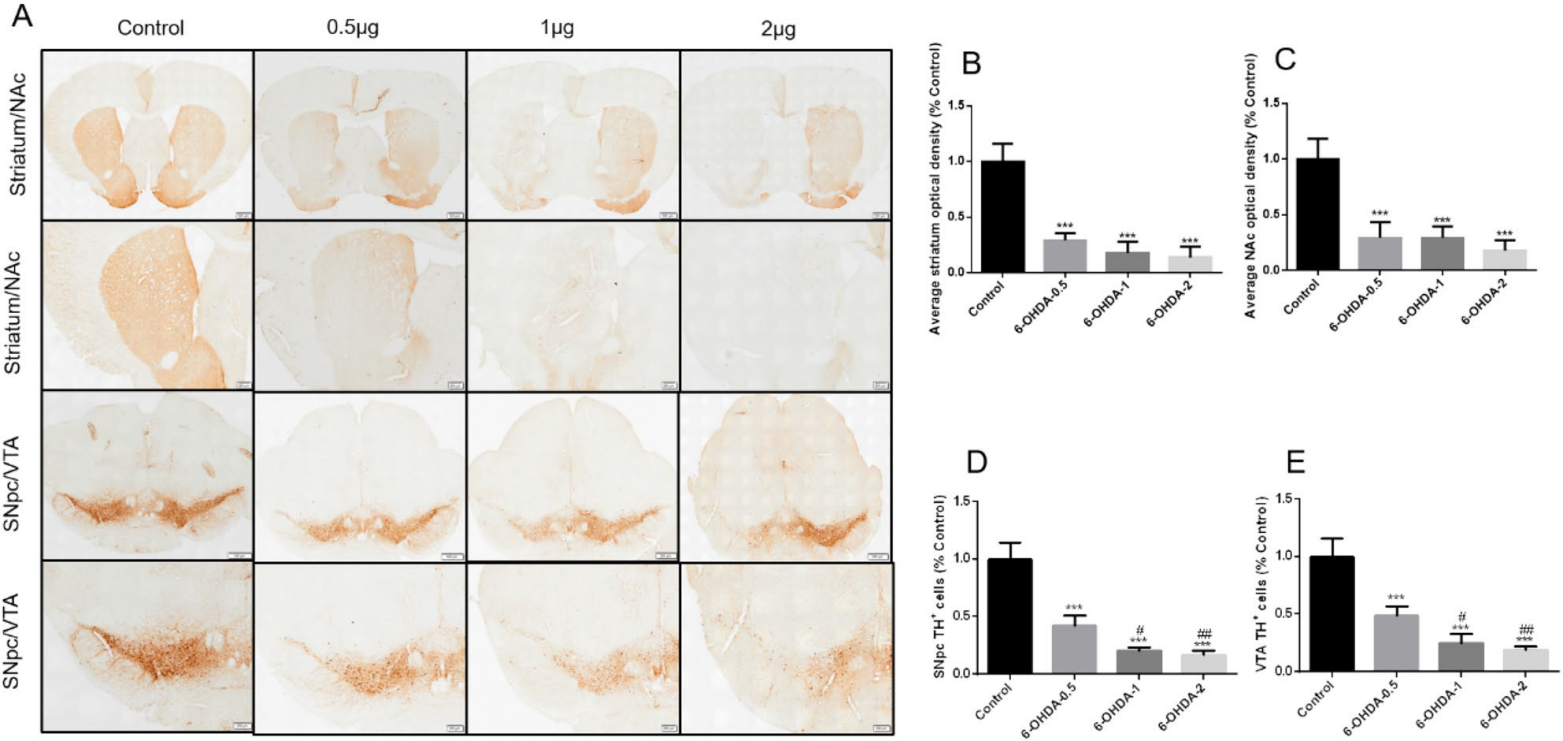
Dose-dependent damage of 6-OHDA in the nigrostriatal system of mice. Representative photomicrographs of brain slices stained for TH^+^ (**A**). Quantification of the striatum/NAc average optical density (% control) and SNpc/VTA TH^+^ cells (% control) were shown (**B**–**E**). ^***^P < 0.001 compared with control, ^#^*P* < 0.05 compared with 6-OHDA-0.5, ^##^*P* < 0.01 compared with 6-OHDA-0.5, n = 4, upper panel scale bars = 500 μm, larger version scale bars = 200 μm.

For histological evaluation, we investigated whether different 6-OHDA doses could damage the nigrostriatal pathway to different extent, and TH^+^ immunohistochemistry in the striatum, NAc, SNpc and VTA was performed. Figure 2A–E shows representative examples and statistical results of successful lesions in all these regions. We found that 6-OHDA lesion caused a significant reduction of both TH^+^ dopaminergic fibres and neurons in the ipsilateral striatum/NAc and SNpc/VTA, respectively. In SNpc/VTA, 1 μg and 2 μg group 6-OHDA further reduced the number of TH^+^ cells compared with 0.5 μg group.

## Motor behavioural assessment of 6-OHDA lesions

**Figure 3:**
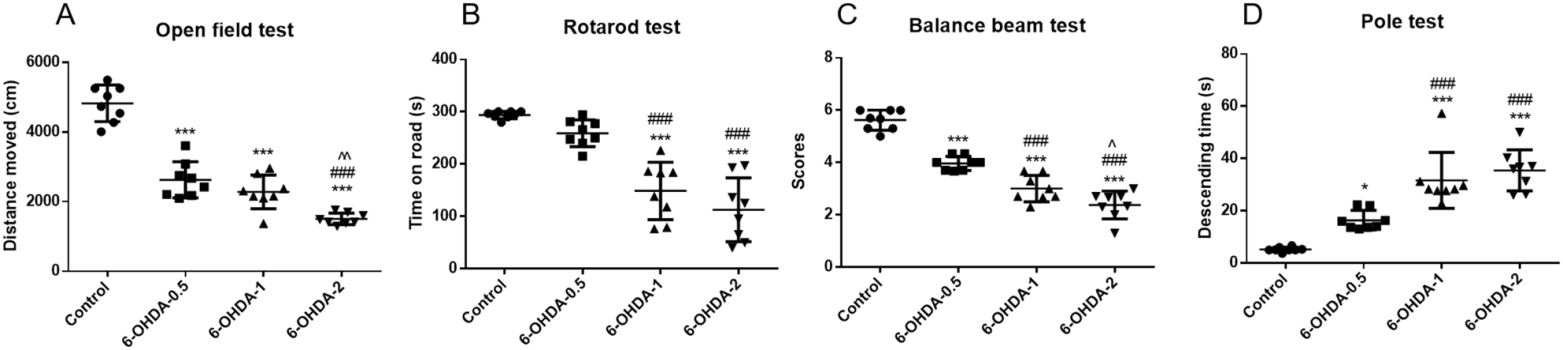
Motor behavioural assessment of 6-OHDA degree of lesion. Different doses of 6-OHDA reduced the total distance traveled by mice in the open field test (**A**) Different doses of 6-OHDA caused significant impairment of motor coordination in the rotarod test in mice (**B**). Different doses of 6-OHDA reduced the score of mice in the balance beam test (**C**). Different doses of 6-OHDA increased the time of mice in pole test (**D**).^***^P < 0.001 compared with control, ^###^*P* < 0.001 compared with 6-OHDA-0.5, ^^^*P* < 0.05, ^^^^*P* < 0.01 compared with 6-OHDA-1, n = 8.

Next, we examined whether damage to the nigrostriatal system by different doses of 6-OHDA could cause various behavioral deficits. Open filed test evaluates the autonomous behavior and exploration behavior of animals in new and different environments. We observed significantly impaired locomotor activity in 0.5 μg and 1 μg 6-OHDA lesioned mice, which was much severer in 2 μg 6-OHDA lesioned animals (Fig. 3A). The rotarod test is used to assess balance, grasping, and motor coordination in mice. Interestingly in the rotarod test, only a high dose (1 μg and 2 μg), rather than 0.5 μg of 6-OHDA reduced the time spent on the rotarod compared to the control group (Fig. 3B). Balance beam test to evaluate the fine motor and sensorimotor functions of mice and the pole test was used to evaluate the motor retardation in mice. Impaired coordination and skilled motor functions in 6-OHDA-lesioned animals were evidenced by dose-dependent decreased scores on the balance beam test (Fig. 3C) and increased time from top to bottom in the pole test (significant in 0.5 μg group and longer in 1 μg and 2 μg group) (Fig. 3D).

## Anxiety- and depressive-like behavior assessment of 6-OHDA lesions

**Figure 4:**
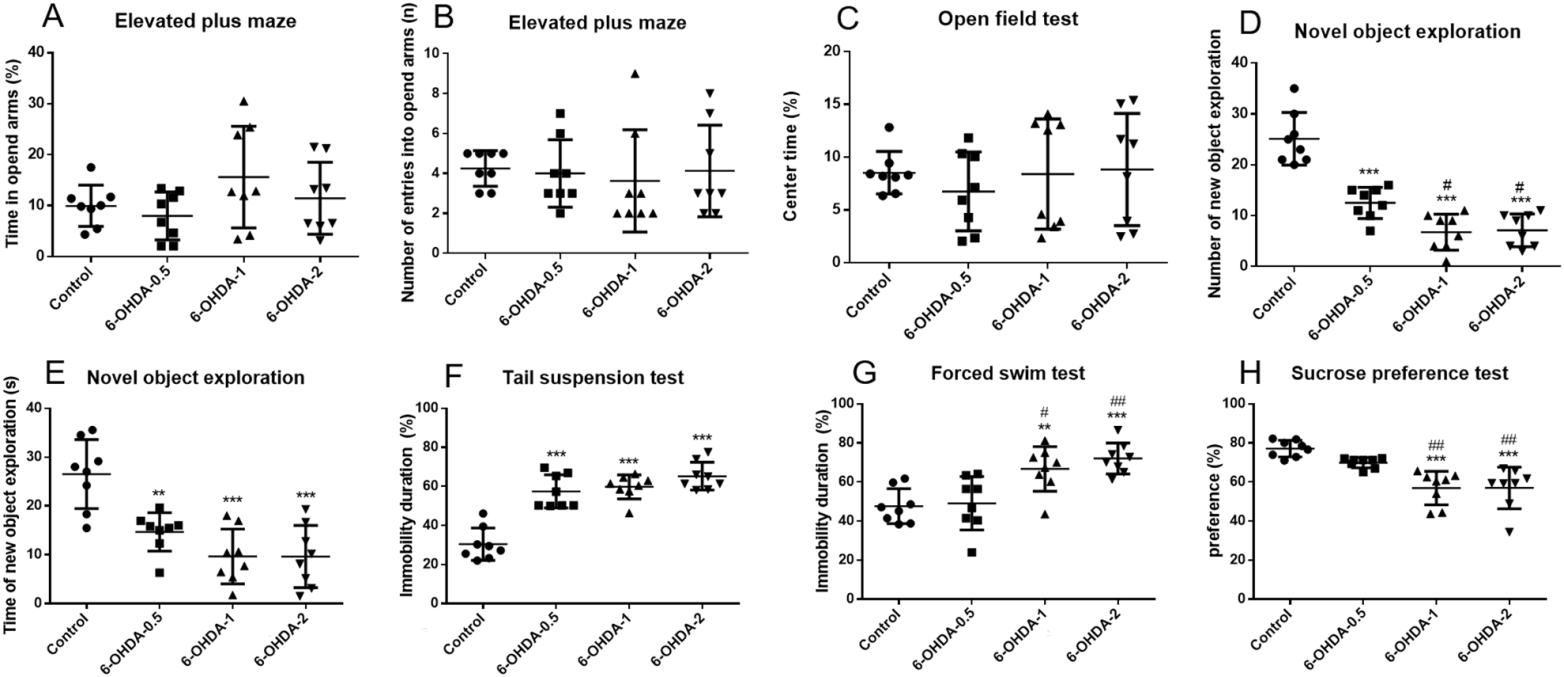
Anxiety- and depressive-like behavior assessment of 6-OHDA degree of lesion. 6-OHDA did not change the time and number of mice entering the open arm in the elevated plus maze test (**A**, **B**). 6-OHDA did not change the time of open-field test in the central area of mice (**C**). 6-OHDA reduced the number and time of mice exploring new objects in novelty exploration test (**D**, **E**). 6-OHDA increased the immobility time of mice in tail suspension test and forced swimming test (**F**, **G**). 6-OHDA reduced the preference of mice to sucrose in the sucrose preference test (**H**). ^**^P < 0.01, ^***^P < 0.001 compared with control, ^#^*P* < 0.05, ^##^*P* < 0.01 compared with 6-OHDA-0.5, n = 8.

In addition to classical motor symptoms, PD patients exhibit non-motor symptoms such as anxiety and depression, and these non-motor phenotypes have been less studied in 6-OHDA PD animal models. The elevated plus maze was used to assess anxiety-like behaviors caused by 6-OHDA lesions. Compared with the control group, 6-OHDA lesion mice did not show significant differences in the time spent in the open arm, and the number of times the mice entered the open arm (Fig. 4A,B). In open field test, we found that there was no difference in the time in the center area (Fig. 4C). This suggests that 6-OHDA does not affect anxiety behavior in mice in this model. This is in accordance with a previous study^19,48^, although there is other studies reported 6-OHDA resulted in impaired anxiety-like behaviors^49,50^. These data suggests whether anxiety is directly related to dopaminergic degeneration remains controversial. We supposed the immobility time of 6-OHDA-lesioned animals in the elevated plus maze and open field experiments confounds the interpretation of the results.

As we know, motor behaviors depend not only on motor skills, but also on exploration motivation. Novel object exploration is to study the exploration behavior of animals to novelty, which is used to assess whether animals are depressed. The tail suspension test and forced swimming are also the most widely used to evaluate the depressive-like behavior in rodents. 6-OHDA reduced the number of explored novel objects in the mice, compared with 0.5 μg, high dose of 6-OHDA lesion mice were further reduced (Fig. 4D). Times of exploring new objects were also reduced, although comparable in different groups (Fig. 4E). 6-OHDA lesion significantly increased the immobility time in the tail suspension test and forced swimming test. In the tail suspension test, all three doses of 6-OHDA increased immobility time, and there was no difference between each group (Fig. 4F). In the forced swimming test, no changes were observed in 0.5 μg group, the immobility time of mice with 1 μg or 2 μg 6-OHDA were further increased (Fig. 4G). Anhedonia is a key feature of depressive-like behavior and often tested by the sucrose preference test. No changes were observed in 0.5 μg group, while 1 μg or 2 μg 6-OHDA significantly decreased the sucrose preference in the test (Fig. 4H), indicating depressive behaviors.

## Gait analysis in 6-OHDA lesioned mice

**Figure 5:**
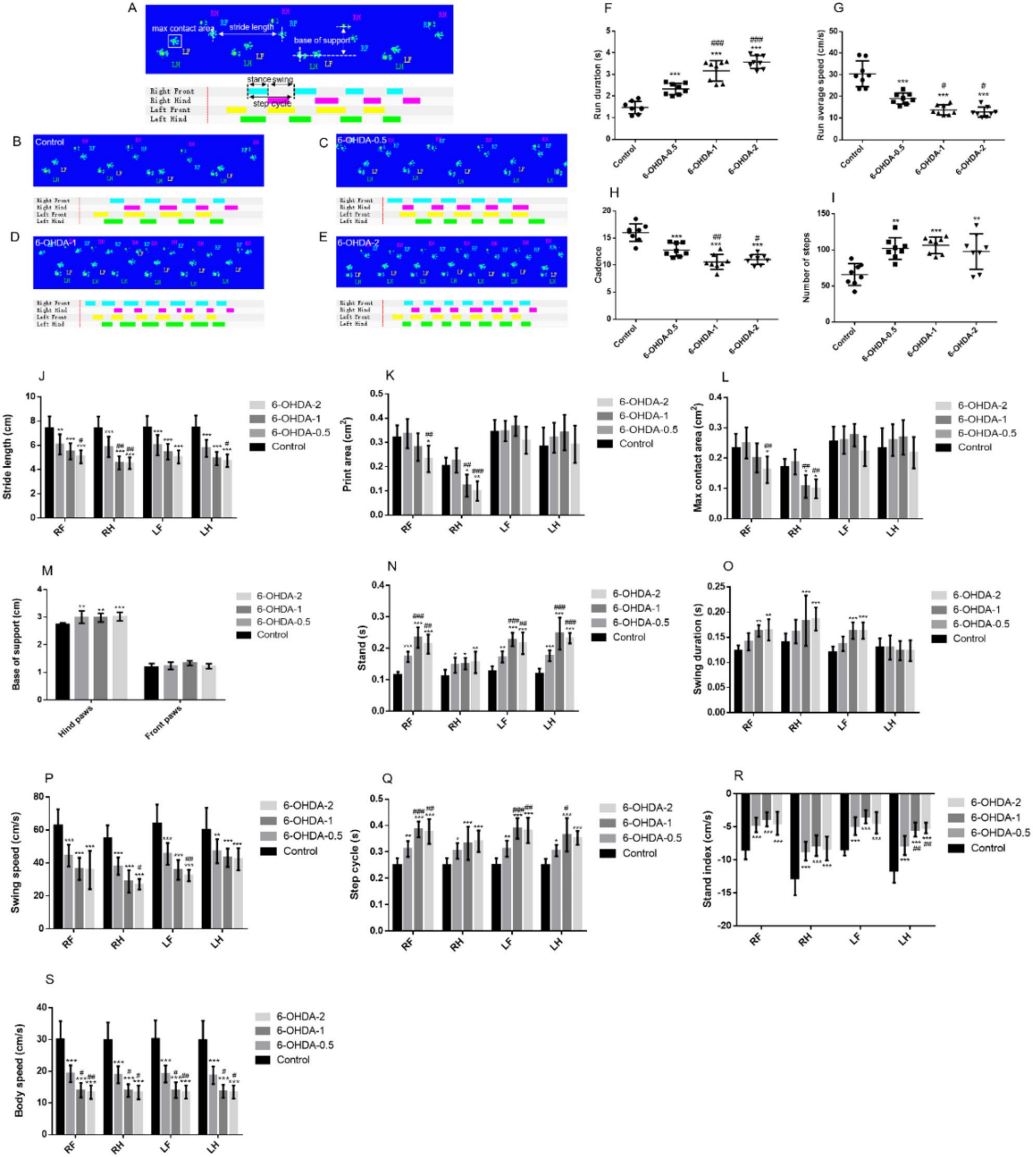
Gait parameters of 6-OHDA mice. Graphical representation of gait parameters. The upper panel (green prints in blue background) shows the digitized prints, while the lower panel (with colorful phase lags) shows the stance phase duration of each individual paw in a single step cycle (**A**). Blue, RF; red, RH; yellow, LF; green, LH. Four walking patterns including control (**B**), 6-OHDA-0.5 (**C**), 6-OHDA-1 (**D**), or 6-OHDA-2 (**E**) group. 6-OHDA increased the run duration and number of steps in the gait analysis of mice (**F**, **I**). 6-OHDA reduced the run average speed and cadence in gait analysis of mice (**G**, **H**). 6-OHDA reduced the stride length of limbs in gait analysis of mice (**J**). 6-OHDA reduced the print area and max contact area of the right limb in the gait analysis of mice (**K**, **L**). 6-OHDA increased the base of support of hind limbs in gait analysis of mice (**M**). 6-OHDA increased the stand, swing duration and step cycle of limbs in gait analysis of mice (**N**, **O**, **Q**). 6-OHDA reduced the swing speed, stand index and body speed of the limbs in the gait analysis of mice (**P**, **R**, **S**).^**^P < 0.01 compared with control, ^***^P < 0.001 compared with control, ^#^*P* < 0.05 compared with 6-OHDA-0.5, ^##^*P* < 0.01 compared with 6-OHDA-0.5, ^###^*P* < 0.001 compared with 6-OHDA-0.5, n = 8.

Gait analysis is a sensitive indicator to detect the movement impairment in PD rodent models. Consistent with CatWalk reports in rats^33,51^, the general, static and dynamic gait parameters changed in unilateral 6-OHDA lesioned mice, and Fig. 5A–E provides a brief overview of gait and gait patterns for the different doses. The detailed classification and definition are illustrated in Table 1. Compared with the sham mice, the mice treated with 6-OHDA had longer time and slower speed of passing through the glass plate (Fig. 5F,G), which was significant in 0.5 μg group and more remarkable in 1 μg or 2 μg groups. Significant reductions in print area and max contact area were only seen in the right paws with higher doses and are suggestive of changes in limb strength and weight-bearing (Fig. 5K,L). This asymmetric presentation in the affected side is due to the unilateral depletion of DA in the contralateral hemisphere, and accordingly exhibited difficulty in balance maintaining. Muscle stiffness and motor dysfunction led to a significant reduction in the stride length and swing speed of the limbs of PD mice, with more reduction in 2 μg group (Fig. 5J,P). Although the duration of the left hind swing phase was similar, the distance of paw movement was reduced, so the speed was reduced. That is, each step a mouse takes, its body moves shorter distances over a longer period of time. Which is similar to the clinical symptoms of PD patients, i.e., they usually take smaller steps and walk more step numbers when attempting to walk. The higher dose did not affect more compared to 0.5 μg, since the numbers of steps were similarly increased with different doses (Fig. 5H), although cadence were slightly further decreased in 1 μg and 2 μg groups (Fig. 5I). The spatial parameters of MFB injured hind limb base of support increased significantly regardless of dose, which is related to the more sensitive role of hind limb in balance instability, greater base of support may compensate for unstable gait and prevent falling (Fig. 5M). Increased in stand, stand index, body speed, step cycle, swing duration, and single stance were observed across all paws (Fig. 5N–S). These dynamic impairments are thought to model bradykinesia seen clinically in PD patients.

**Table 1:** Classification and definition of gait parameters.

Parameter	Definition
Run average speed	The average speed of animals run from entering until leaving the walkway
Run duration	The time between first print and last print of the walkway
Cadence	The number of footsteps have taken per unit time
Number of steps	The total number of footprints in walkway
Stride length	The distance (cm) between successive placements of the same paw. This parameter represents the relative spatial relationships between paws
Print area	The area (cm^2^) of the complete impression of the claw heart and toe each time the paws contact the glass plate. This parameter describes the surface area in complete print
Max contact area	The maximum area (cm^2^) of a paw that comes into contact with glass plate. This parameter is used to describe the maximum contact area between each paws and the running platform form contact to departure
Base of support	The average width (cm) between either the front paws or the hind paws. It is derived by measuring the distance (cm) between the mass mispoints of the two forelimb or hind limb prints at the maximum contact. This parameter in an indicator of double-limb support
Stand	The duration (s) of the paw contact with the glass plate in a step cycle
Swing duration	The duration (s) of no contact between the same paw and ground during a step cycle
Swing speed	The speed (cm/s) of the paw during swing phase. This parameter refers to the velocity of the moving limb during the swing phase. It is computed form stride length and swing duration
Step cycle	The time (s) between two consecutive initial contacts of the same paw with the glass plate and consists of the stand phase and swing phase
Stand index	The speed (cm/s) when the paw loses contact with the glass plate at the initiation of the swing phase
Body speed	The body speed of a paw during a step cycle

## Mitochondrial structure in striatum and SNpc of 6-OHDA lesioned mice

**Figure 6:**
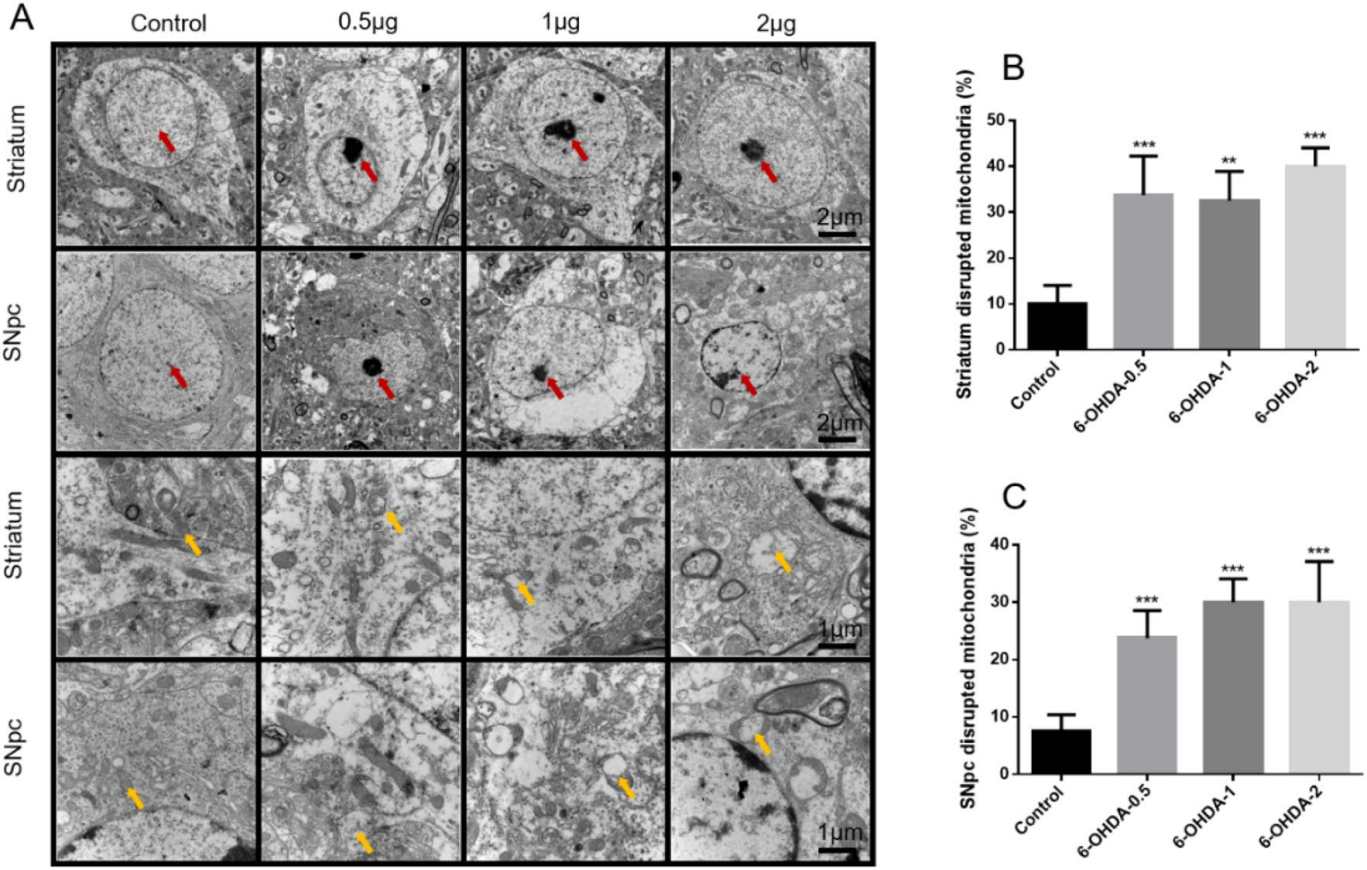
TEM analysis of mitochondria in the striatum and SNpc regions of from 6-OHDA or control. The representative images are shown and red arrows indicate nucleus, yellow arrows indicate mitochondria (**A**). Quantification of the striatum and SNpc disrupted mitochondria (%) were shown (**B**, **C**). ^**^P < 0.01 compared with control,^***^P < 0.001 compared with control, n = 4.

Mitochondrial dysfunction is an important feature of PD^52,53^. We studied the striatum and SNpc mitochondrial changes of mice treated with different doses of 6-OHDA through TEM. Figure 6A–C shows representative examples and statistical results of successful lesions in all these regions. The red arrow points to the nucleus, and there is no change in the nucleus morphology of the striatum and SNpc of control mice, and chromatin condensation occurs after 6-OHDA treatment. The yellow arrows indicate mitochondria. The striatum and SNpc neurons of mice in the control group had a large number of dendrites and dense and complete mitochondrial structure. The distinctive morphological feature of 6-OHDA-treated was changed in mitochondria that the morphology was fragmented, the ridge was destroyed, and the cavitation was obvious. These results indicated that 6-OHDA treatment showed characteristic morphological features related to apoptosis.

## Glial cell activation in striatum and SNpc of 6-OHDA lesioned mice

**Figure 7:**
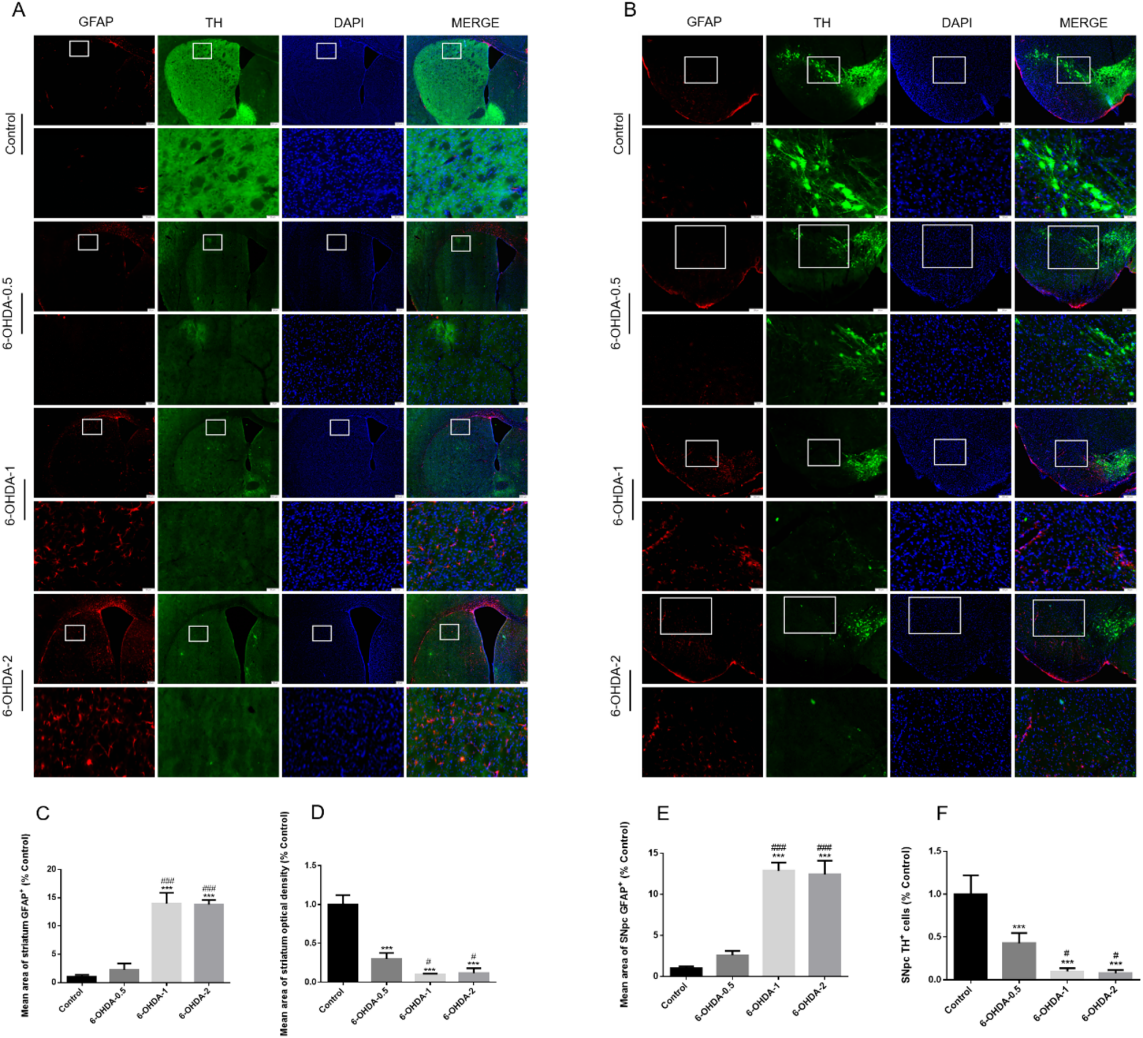
The activation of astrocytes in striatum or SNpc after MFB injection 6-OHDA. Immunofluorescence representative micrographs showed labelling of TH^+^ (green) and of GFAP^+^ from the striatum and SNpc of 6-OHDA-lesioned mice (**A**, **B**). 6-OHDA increased GFAP^+^ (red) and decreased TH^+^ (green) in the striatum and SNpc of mice. Quantification of the striatum or SNpc coverage by GFAP^+^ or TH^+^ staining were shown (**C**–**F**). ^*^P < 0.05, ^**^P < 0.01, ^***^P < 0.001 compared with control, ^#^*P* < 0.05 compared with 6-OHDA-0.5, ^###^*P* < 0.001 compared with 6-OHDA-0.5, n = 4, upper panel scale bars = 200 μm, larger version scale bars = 50 μm.

**Figure 8:**
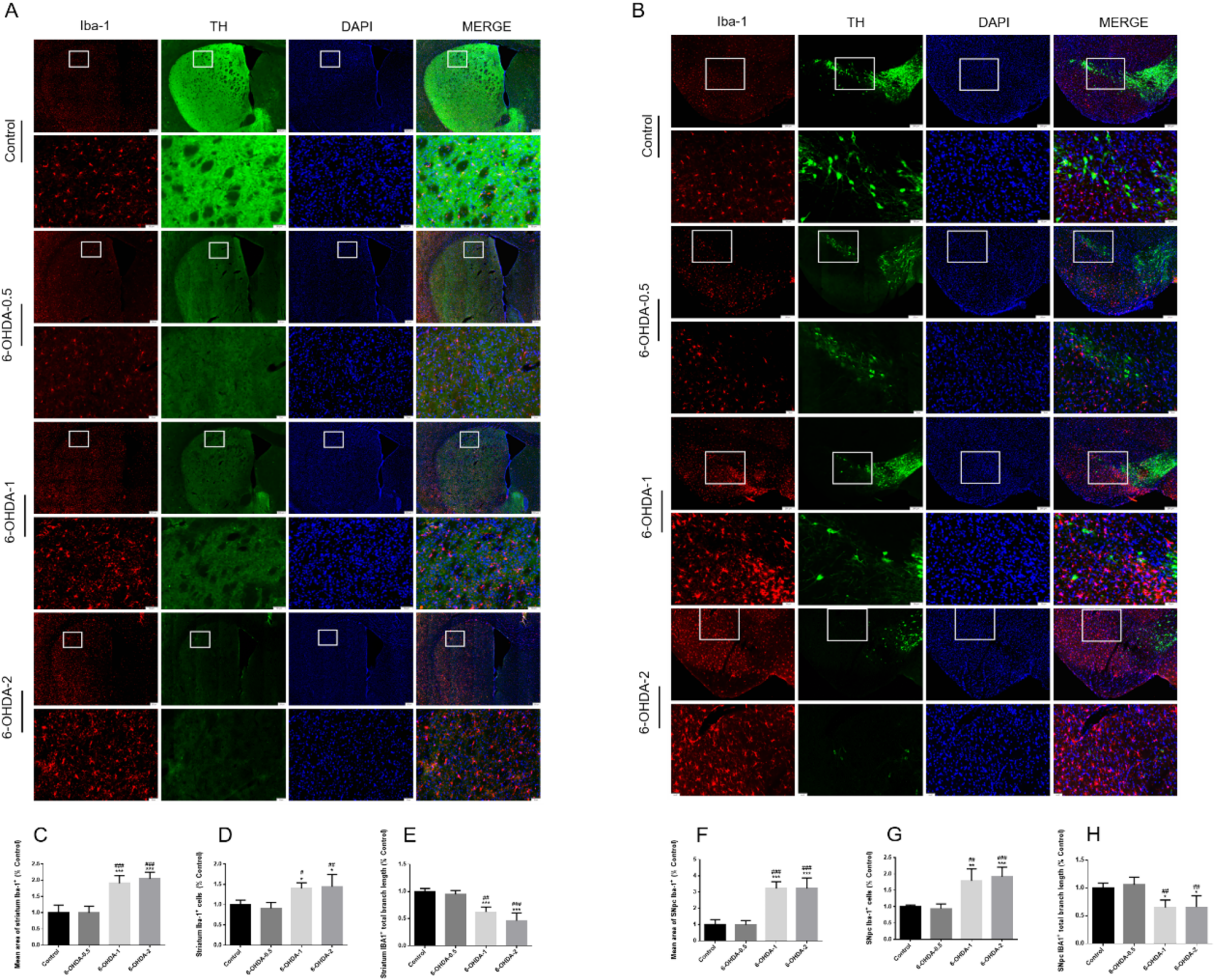
The activation of microglial in striatum or SNpc after MFB injection 6-OHDA. Immunofluorescence representative micrographs showed labelling of TH^+^ (green) and of Iba-1^+^ from the striatum and SNpc of 6-OHDA-lesioned mice (**A**, **B**). 6-OHDA increase the mean area of Iba-1^+^ and Iba-1^+^ cells as well as decrease Iba-1^+^ total branch length in the striatum and SNpc of mice. Quantification of the striatum or SNpc mean area of Iba-1^+^, Iba-1^+^ cells and Iba-1^+^ total branch length were shown (**C**–**H**). ^*^P < 0.05, ^**^P < 0.01, ^***^P < 0.001 compared with control, ^#^*P* < 0.05 compared with 6-OHDA-0.5, ^##^*P* < 0.01 compared with 6-OHDA-0.5, ^###^*P* < 0.001 compared with 6-OHDA-0.5, n = 4, upper panel scale bars = 200 μm, larger version scale bars = 50 μm.

Neuro-inflammation mediated by glial cells is described as a common feature of PD and is believed to further trigger the progression of neurodegenerative events^54^. We characterized astrocytes and microglia in the striatum and SNpc regions of 6-OHDA mice. As shown in Fig. 7 the mean area of of GFAP^+^ astrocytes indicated by fluorescence intensity was comparable in both the striatum and SNpc, suggesting 1 μg or 2 μg 6-OHDA injection induced robust astrocytes activation. However, there was no significant activation in 0.5 μg group (Fig. 7C,E). Similarly, there was no changes of the number and mean area of Iba-1^+^ cells in 0.5 μg group, while 1 μg or 2 μg 6-OHDA significantly increased both the number and mean area, implying microglia activation (Fig. 8A–D, F,G). Accordingly, a decreased length of total Iba-1 branches was observed in both the striatum and SNpc (Fig. 8A,B,E,H), further supporting an activated phenotype. We also observed significant loss of TH^+^ fibers in the striatum and a decrease in fluorescence intensity of TH^+^ neurons in the substantia nigra of mice treated with 6-OHDA, compared with 0.5 μg, high dose of 6-OHDA lesion mice were further increased (Fig. 7D,F).

## Discussion

Rodents animal models play an important role in drug development. They are used to test compounds before applying them in clinical trials in order to evaluate potential efficacy for a specific disease and minimize risks for humans. In that context accurate and reproducible behavioral tests in animal models are of major importance in the development and evaluation of new therapies for central nervous system disease. MFB impairment is one of the most common PD models and the use of this model in mice has been strongly criticized in the past due to high mortality. In our study, the weight of intensive care mice gradually recovered on the 14 day after implementation, and the survival rate reached 100%. During this period, moist food should be provided twice a day, along with a subcutaneous injection of 1 ml sodium lactate Ringer's solution to monitor and prevent dehydration. We have noticed that the prevalence of penile prolapse in mice undergoing 6-OHDA surgery has increased. In order to limit this postoperative complication, we applied local antibiotics to the penis of animals showing signs of prolapse, which was consistent with the recent reports^35,55–57^.

Previous studies of 6-OHDA injections in mouse MFB have either been too high in doses or examined only motor behavior^35,36^. Here, we choose 0.5,1 and 2 μg/μL 6-OHDA to established a partial MFB impairment model in PD mice with different doses of 6-OHDA and characterized it by motor behavior, non-motor behavior as well as gait analysis. Drug-induced rotation and non-drug-induced behavioral tests based on lateral sensorimotor integration and forelimb dyskinesia were strongly associated with the extent of nigrostriatal lesions^35,58^. And over time, the damage produced by 6-OHDA is relatively stable and does not recover spontaneously. We observed significantly impaired locomotor activity in the 6-OHDA-lesioned group, which was more severe in the higher dose lesioned animals, as indicated by reduced locomotor activity in the open field test, dragging the lower part of the body so that the body could walk closer to the ground, an abnormal gait, and exhibiting backward positioning of the lesioned hind limbs^59,60^. Interestingly in the rotarod test, 0.5 μg 6-OHDA did not change the time mice spent on the rotarod compared to the control group, and we guess that low-dose 6-OHDA did not affect their ability to learn. In addition to classical motor symptoms, PD patients exhibit non-motor symptoms such as anxiety and depression, and these non-motor phenotypes have been less studied in 6-OHDA PD animal models. To gain further insight into the behavioral effects of MFB dopaminergic injury, we assessed the presence of anxiety- and depression-like behaviors in this model, but whether anxiety is directly related to DA degeneration remains controversial^19,48–50^. One study reported that dorsal striatum injection of 6-OHDA resulted in impaired gait dynamics in mice with olfactory deficits and depression- and anxiety-like behaviors, but another study showed that bilateral MFB lesions had no effect on anxiety. This is because the immobility time of 6-OHDA-lesioned animals in the elevated plus maze and open field experiments confounds the interpretation of the results. Given our results and findings, it does not appear that anxiety is a common feature of this PD model. As we all know, motor activity depends not only on motor skills, but also on exploration motivation. The dorsal striatum is crucial for motor control and motor learning, while the ventral striatum, especially NAc, is the key structure for emotion and motivation processing, regulation and reward system, and serves as the edge-motor interface^61,62^. Although MFB lesions mainly affect dopaminergic neurons of the SNpc, they also lead to a decrease in VTA dopaminergic neurons, which constitute the mesolimbic pathway and innervate the NAc.

To date, several methods have been described to determine gait functions in animal models of PD and each are associated with its own strengths and weaknesses. For example, the treadmill locomotion test used to assess locomotor activity can be used to evaluate parameters such as walking speed, swing and stance time^63^. The cylinder test used to evaluate the asymmetry of the use of forelimbs or the absence of kinesia. Based on the exploration behavior of rats and the exploration environment of limbs, general sports injuries can be evaluated, but limited by manual scored and restricted to forelimbs only^64^. Other tests used to evaluate forced motion, such as rotarod, allow for investigation of more dynamic gait parameters, but do not analyze specific changes in walking patterns^38^. Footprint analysis and stepping tests allow determination of stepping patterns and forepaw adjusting steps^55^, but these tests have limitations in that the researcher involved in the experiment either has to hold the animal (stepping test) or have to examine the motor behavior which creates subjectivity. In addition, footprint analysis and stepping test focus exclusively on detecting static gait parameters, and to assess a large number of gait parameters researchers have to perform multiple tests. Using these established methods, it is usually necessary to wait until the DA is completely lost to observe the functional damage. It is beneficial to identify reliable signs of functional impairment as early as possible after injury and evaluate potential therapeutic drugs.Here, we studied the time stability and repeatability of gait patterns in mice lesioned by unilateral MFB 6-OHDA through automatic quantitative gait analysis (Catwalk). Consistent with many CatWalk research reports, the general, static and dynamic gait parameters of animals changed after 6-OHDA MFB infusion. The CatWalk system can provide a reliable and objective standard for the stratification of gait changes. These findings have prospects in the research of PD disease progress and new treatment methods.

The current view is that neuro-inflammatory glial cell activation, mitochondrial dysfunction and oxidative stress are related to the pathogenesis of PD. 6-OHDA produces free radicals and causes mitochondrial dysfunction. The inhibition of complex I and IV mediated by 6-OHDA leads to the release of cytochrome c into the cytoplasm and induces the apoptosis of dopaminergic neurons. 6-OHDA also increases the pro-inflammatory cytokine TNF-α, IFN-γ and IL-6 expression, which mediates dopaminergic neurodegeneration leading to PD symptoms^65,66^. Previous studies have shown that injection of 6-OHDA into MFB will enhance neuro-inflammation, resulting in increased proliferation of microglia and astrocytes in the striatum which is consistent with our study^54,67^. Some studies showed that the most significant changes in the activation of microglia and astrocytes occurred within a few days after 6-OHDA injection, and gradually decreased with time, but other studies showed that the activation state was prolonged, especially in astrocytes^68–70^. A significant increase in the area occupied by astrocyte specific cells GFAP^+^ and an increase in microglia specific cells Iba-1^+^ in the ipsilateral dorsal striatum and SNpc of 6-OHDA-lesioned animals, showing microglial proliferation, a significant decrease in the length of total Iba-1 branches, and phenotypic activation. More and more evidence shows that astrocytes play an important role in the progression of PD^71,72^. Although microglia were previously considered to be the main inflammatory cells in the central nervous system, the inflammatory activation of astrocytes is usually more persistent than microglia, and is considered to be important in the chronic inflammatory activation associated with PD^73^.

Of course, our study have some limitations, such as the experiment was only conducted in male mice, and estrogen was known to affect the degree of lesions, behavioral performance and the neuroinflammation response^74,75^. In addition, we selected wild-type C57BL/6 mice, and in two other studies they selected Rgs5^gfp/+^ reporter mice and bacterial artificial chromosome driven transgenic mice^35,76^. In conclusion, we injected different doses of 6-OHDA into the MFB of mice to mimic the progressive stages of human PD by inducing various degrees of nigrostriatal DA loss, to develop a graded animal model of PD, and to characterize each stage of motor behaviors as well as anxiety-like and depression-like behaviors. We report for the first time the analysis of gait in this PD mouse model using an automated gait analysis system, showing that the 6-OHDA-induced MFB mice model is more suitable for studying gait dysfunction. Finally, we observed the effects of different doses of 6-OHDA on mitochondria of mice substantia nigrostriatum neurons, we also detected the reactive glial reaction in the lesion area of the substantia nigra striatum, which provides a certain experimental basis for the conversion of glial cells into neurons. In mimic different stages of PD, 0.5 μg 6-OHDA injection in mice induced certain symptoms in motor behaviors (such as data from gait analysis), while unobvious changes in other parameters (such as data from rotarod test). There were no non-motor symptoms and no obvious glial activation in the nigrostriatal pathway at this moment, although there was a specific neuronal loss and mitochondrial disruption. However, behavioral, as well as nigrostriatal neuropathological manifestations were largely consistent in 1 μg and 2 μg 6-OHDA groups, indicating that 1 μg lesion is already sufficient to mimics an advanced stage in PD. We thought 0.5 μg was similar to the early stages of PD with moderate/not too serious lesions, while 1 μg 6-OHDA was necessary and sufficient to induce both motor and non-motor symptoms in late stage. Therefore, we believe different doses of 6-OHDA injection in MFB of mice is a powerful tool to mimic the different stages of the disease.These findings have prospects in the research of PD disease progress and new treatment methods.

## Data Availability

All data in our study are available upon request from the corresponding authors.
